# Intracranial Rosai-Dorfman Disease: A Case to Remember

**DOI:** 10.7759/cureus.32605

**Published:** 2022-12-16

**Authors:** Bhavik S Unadkat, Shivali V Kashikar, Pratik J Bhansali, Neha D Shetty, Sheetal S Shelar, Manasa Suryadevara, Abhijay Dharmadhikari

**Affiliations:** 1 Radiodiagnosis, Datta Meghe Institute of Medical Sciences, Wardha, IND; 2 Pathology, Datta Meghe Institute of Medical Sciences, Wardha, IND

**Keywords:** rosai-dorfman disease, pet ct scan, sinus histiocytosis with massive lymphadenopathy, neuro radiology, intracranial neoplasms, benign lymphoproliferative, hemophagocytic lympho-histiocytosis

## Abstract

Sinus histiocytosis with massive lymphadenopathy (SHML), an alternative term for Rosai-Dorfman disease (RDD), is a rare benign idiopathic immune-related lymphoproliferative condition. The central nervous system (CNS) has been documented to be involved in RDD, although lymph nodes are the organs that are most frequently and primarily associated with the disease manifestation. Nonetheless, CNS involvement in RDD is rare and poorly understood. As a result, there is a lack of a solid basis for therapeutic approaches for CNS involvement in RDD. Here, we present a case of RDD with cerebral involvement, a rare presentation of RDD with atypical symptoms. A brief assessment of the radiographic appearance, histological findings, and the peculiar manifestations of the disease is provided.

## Introduction

Sinus histiocytosis with large lymphadenopathy, another name for Rosai-Dorfman disease (RDD), is a rare benign condition that is characterized histologically by lymphatic sinus dilatation brought on by histiocyte proliferation. Destombes provided the first description of the illness [1]. It was followed by the identification as a separate clinicopathologic entity by Rosai and Dorfman [2]. The condition primarily affects the younger population with male preponderance [3]. RDD frequently progresses slowly and is recurring in nature. RDD patients typically present with symptoms such as fever and cervical lymphadenopathy and raised white blood count on blood investigations. The emperipolesis, in which histiocytes phagocytize lymphocytes, plasma cells, erythrocytes, or polymorphonuclear leukocytes, is the distinctive histopathologic hallmark.

## Case presentation

Presentation and examination

A 55-year-old male presented with complaints of left-sided hemicranial headache on the frontotemporal region associated with bilateral nasal blockage for two years. These symptoms were insidious in onset and gradual in progression, which aggravated for the past three months. The patient initially had a nasal blockage on the left side, which progressed towards the right side of the nostril leading to anosmia. The nasal stuffiness did not relieve on any maneuvers or medications and compelled the patient to restore to mouth breathing. He also had a bilateral decreased hearing and associated complaints of left-sided photophobia. The patient had also noticed a change in the tone of voice over the course of two years. There were no complaints of dysphagia, odynophagia, epistaxis, or seizures. No other ear, nose, and throat complaints were reported. The patient had no past history of hypertension, diabetes, tuberculosis, thyroid disorders, blood disorders, genetic disorders, or any other endocrine disorders. The patient also did not report any use of any medications. For these above-mentioned complaints, the patient presented to our tertiary care center and was thus advised further work-up. On examination, bilateral ears were within normal limits. A nasal examination revealed a reddish mass occupying the left nasal cavity with no associated external deformity of the nose. Indirect laryngoscopy revealed no obvious abnormality. No evidence of palpable lymph nodes was detected. The patient was therefore advised to undergo basic hematological investigations along with magnetic resonance imaging (MRI) brain with contrast, positron emission tomography and computed tomography (PET-CT), and biopsy from the nasopharyngeal region.

Laboratory findings

**Figure 1:**
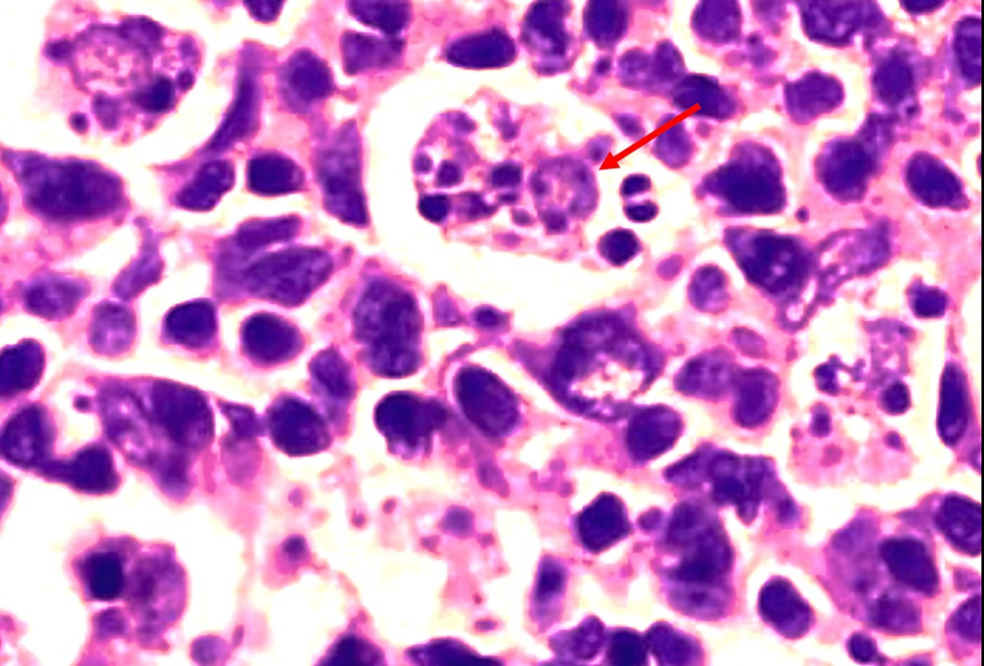
Histopathology of sinonasal mass biopsy specimen Hematoxylin and eosin stain smear showing a macrophage with lymphocytic emperipolesis (red arrow).

The complete blood count investigation revealed decreased hemoglobin (7.9 g/dL), and the peripheral smear showed hypochromic anemia. There was raised white blood cell count (18,000/mcL), suggesting leucocytosis; the erythrocyte sedimentation rate was found to be 48 mm/hr. Biopsy obtained from the nasopharyngeal region revealed emperipolesis. There was also evidence of dense infiltrates of plasma cells and histiocytes. No areas of granuloma or necrosis were seen (Figure 1).

Imaging findings

**Figure 2:**
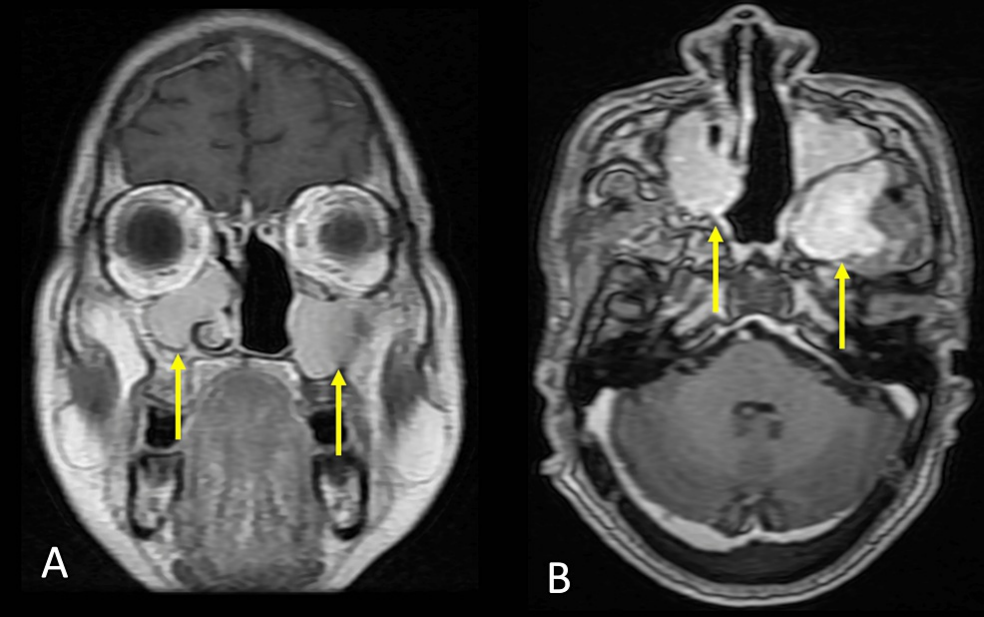
Magnetic resonance imaging with contrast A. Coronal contrast section showing sinonasal involvement (yellow arrow). B. Axial contrast section showing bilateral maxillary sinus, left sphenoid sinus involvement (yellow arrows).

The patient further underwent an MRI of the brain with contrast which revealed a large well defined T1 hypointense, T2 hyperintense lesion with few flow voids measuring 7.5 x 4.5 x 7.8 cm (AP x TR x CC) in the nasopharynx, left nasal cavity obliterating bilateral fossa of Rosenmüller. The mass was noted to extend anteriorly into the nasal cavity superiorly; there was an intracranial extension into anterior cranial fossa planum sphenoidale and anterior clinoid process, inferiorly there was the involvement of the left middle turbinate, osteomeatal complex causing its widening and its extension up to oropharynx (Figure 2).

**Figure 3:**
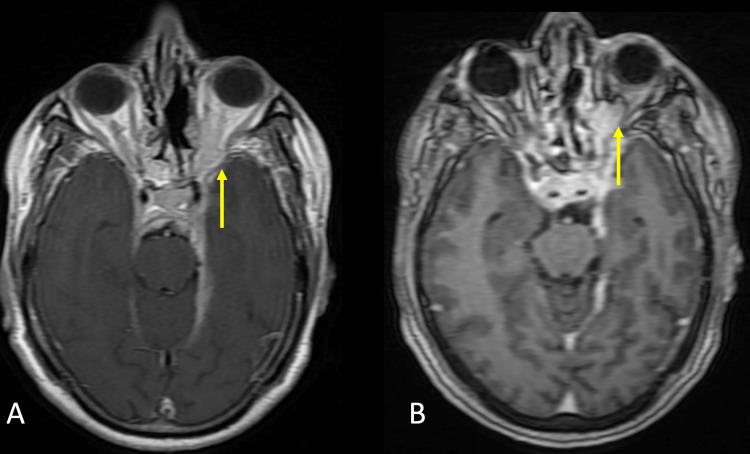
Magnetic resonance imaging with contrast A. Axial T1WI fast spin echo contrast sections with a yellow arrow pointing at left orbital involvement. B. Axial T1WI contrast section with a yellow arrow pointing at left orbital involvement.

The mass was along ventral pons and tentorial leaflet on the left side. Subtle enhancement in the intracanalicular segment of the left VII-VIII nerve complex was noted. The lesion involved the left infratemporal fossa and left pterygopalatine fossa crossing midline and involved the right maxillary sinus. The lesion was also extending laterally into the left orbit medial and inferior quadrants extending to involve medial and inferior recti muscles (Figure 3).

**Figure 4:**
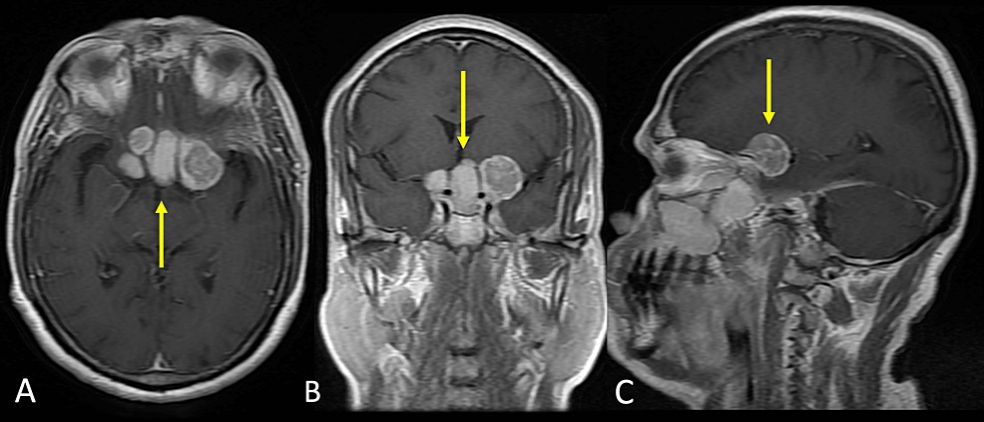
Magnetic resonance imaging with contrast images A. Axial T1WI contrast section showing multiple heterogeneously enhancing mass in the supra and parasellar region suggesting intracranial involvement of disease (yellow arrow). B. Coronal T1WI contrast section showing multiple heterogeneously enhancing mass in the supra and parasellar region suggesting intracranial involvement of disease (yellow arrow). C. Sagittal T1WI contrast section showing heterogeneously enhancing mass in the suprasellar region suggesting intracranial involvement of disease (yellow arrow).

It demonstrated intracranial involvement into the suprasellar region and did not show restricted diffusion. It involved both cavernous sinuses posteriorly (Figure 4).

**Figure 5:**
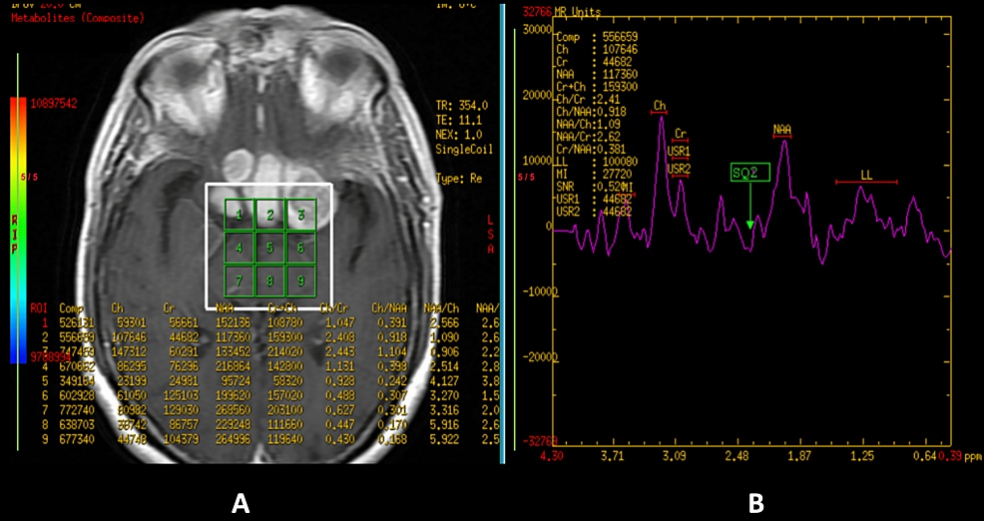
Magnetic resonance imaging spectroscopy A. Contrast-enhanced magnetic resonance imaging of the brain with voxel placement in the region of interest. B. Magnetic resonance imaging spectroscopy suggesting reduced N-acetylaspartate (NAA) and raised choline with an increased choline-creatinine ratio in the region of interest.

These features were suggestive of a well-defined nasopharyngeal neoplastic lesion with extensions as described. Magnetic resonance (MR) spectroscopy revealed reduced N-acetylaspartate (NAA) and raised choline with an increased choline-creatinine ratio (Figure 5).

**Figure 6:**
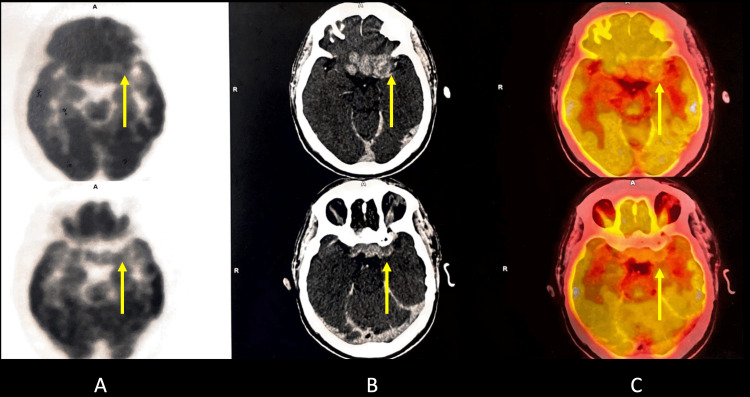
Positron emission tomography-computed tomography scan A. Positron emission tomography-computed tomography scan of the head showing intracranial extension, the intracranial component of metabolically active mass (yellow arrow). B. Contrast-enhanced computed tomography scan of the brain showing intracranial extension, the intracranial component of metabolically active mass (yellow arrow). C. Positron emission tomography-computed tomography scan fusion image showing intracranial extension, the intracranial component of metabolically active mass (yellow arrow).

**Figure 7:**
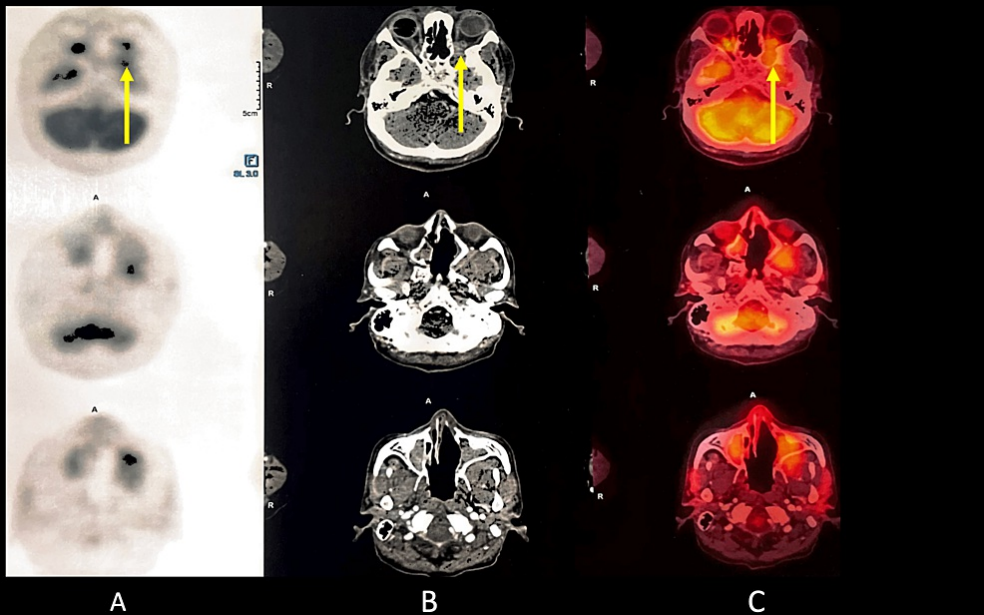
Positron emission tomography-computed tomography scan A. Positron emission tomography-computed tomography scan of the head reveals an ill-defined minimally enhancing mass lesion in the masticator space on the left side, showing low-grade diffuse fluorodeoxyglucose uptake with standardized uptake value max of 6.1 with obliteration of the retroantral fat on the left side. There is a superior extension of the mass reaching up to the apex of the left orbit (yellow arrow). B. Contrast-enhanced computed tomography scan of the head reveals an ill-defined minimally enhancing mass lesion in the masticator space on the left side, showing low-grade diffuse fluorodeoxyglucose uptake with standardized uptake value max of 6.1 with obliteration of the retroantral fat on the left side. There is a superior extension of the mass reaching up to the apex of the left orbit (yellow arrow). C. Positron emission tomography-computed tomography fusion scan of the head reveals an ill-defined minimally enhancing mass lesion in the masticator space on the left side, showing low-grade diffuse fluorodeoxyglucose uptake with standardized uptake value max of 6.1 with obliteration of the retroantral fat on the left side. There is a superior extension of the mass reaching up to the apex of the left orbit (yellow arrow).

**Figure 8:**
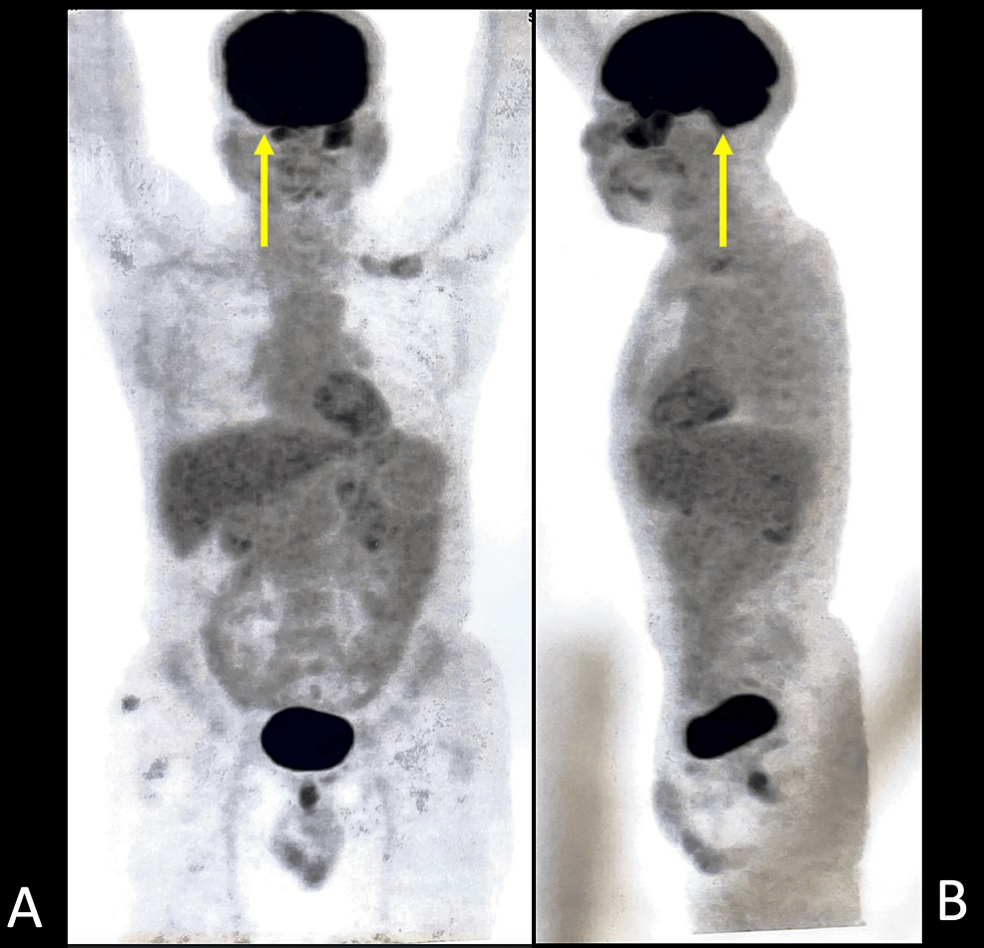
Positron emission tomography-computed tomography scan A. Coronal positron emission tomography-computed tomography scan showing increased metabolic activity in the head region (yellow arrow). B. Sagittal Positron emission tomography-computed tomography scan showing increased metabolic activity in the head region (yellow arrow).

PET-CT of the head revealed an ill-defined minimally enhancing mass lesion in the masticator space on the left side, showing low-grade diffuse fluorodeoxyglucose (FDG) uptake with maximum standardized uptake value (SUVmax) 6.1 with obliteration of the retroantral fat on the left side. There was the superior extension of the mass reaching up to the apex of the left orbit with contiguous intracranial extension; the intracranial component measured about 2.4 x 4.6 cm neck showed non-FDG-avid small bilateral cervical adenopathy. Thorax, abdomen, and pelvis revealed no significant hypermetabolic foci (Figures 6-8).

## Discussion

RDD is statistically found to be quite infrequently occurring non-malignant histiocytic condition. Symptoms generally present include that enlarged and painless cervical lymph nodes. Over forty percent of individuals may experience extranodal involvement, even without accompanying lymphadenopathy. The most prevalent type of extranodal Rosai-Dorfman's illness is cutaneous lesions. Rosai-Dorfman's illness that just affects the skin, on the other hand, is uncommon because it typically manifests as a systemic condition [4,5]. The majority of extranodal locations that are affected are the soft tissue and skin, which is about seventeen percent, and the least affected sites with less than 1 percent of occurrence are the heart, gastrointestinal tract, and breast. Other commonly occurring extranodal sites include the nasal cavity and paranasal sinuses, the eye, orbit, and ocular adnexa, the salivary gland, the oral cavity, the kidney and genitourinary tract, the respiratory tract, the liver, and the tonsil. The incidence of RDD affecting the central nervous system (CNS) was found to be about seven percent, which is a rarity in itself [6]. Conditions that mimic intracranial RDD include plasma cell granulomas, Langerhans cell histiocytosis, granulomatous illnesses, lymphoproliferative disorders, and neurofibromatosis [7-12]. Meningioma appears to be the closest differential to intracranial RDD, which poses a diagnostic challenge to clinicians and radiologists alike. RDD is still thought to be of unknown etiology. Various studies have classified it as a neoplastic, infectious process, or immunological [13,14]. The occurrence of the disease has also been linked with viral etiology. Rosai-Dorfman's illness has no established cure. However, it can be treated with a combined approach, which includes surgery, radiotherapy, and chemotherapy. In situ hybridization has identified Epstein-Barr virus (EBV) and human herpes virus (HHV-6) in certain individuals with RDD. Rosai-Dorfman's illness is an uncommon diagnosis that manifests itself in many ways in imaging studies. Neuroradiological manifestations are commonly multifocal and affect the intracranial compartment, the spine, or the neck, with painless lymphadenopathy occurring most frequently. Unifocal involvement of RDD needs to be screened, and routine follow-up is advised for patients. Involvement in additional body regions may be detected and thus clinically helpful in early detection and prompt treatment. Multifocal involvement can be synchronous or metachronous in nature, thus aiding in swift diagnosis [15].

## Conclusions

RDD-related CNS involvement is a singular and peculiar presentation that needs extensive work-up in order to reach a precise diagnosis. This case report encompasses all the necessary and needful investigations and findings pertaining to intracranial involvement noted in the disease progression. There are no standardized treatment guidelines due to the paucity of RDD-related intracranial manifestations. Surgery is typically the treatment for localized presentations; unfortunately, resection is not always feasible, and other therapeutic approaches may be required for disease management. Thus, a standardized approach to managing patients with RDD is of utmost importance.
